# Exogenous auxin regulates the growth and development of peach fruit at the expansion stage by mediating multiple-hormone signaling

**DOI:** 10.1186/s12870-023-04514-2

**Published:** 2023-10-18

**Authors:** Yanping Zhang, Ziwen Su, Linjia Luo, Pengkai Wang, Xudong Zhu, Jiecai Liu, Chen Wang

**Affiliations:** 1 College of Horticulture, Nanjing Agricultural University, Nanjing, 210095 China; 2 Faculty of Horticultural Science and Technology, Suzhou Polytechnic Institute of Agriculture, Suzhou, 215008 China; 3 Inner MongoliaAgricultural University, Huhehaote, 010010 China

**Keywords:** Auxin; peach, Fruit expansion stage, Multiple-hormone signaling

## Abstract

**Background:**

Fruit expansion stage is crucial to fruit yield and quality formation, and auxin plays a significant role by mediating multi-hormone signals during fruit expansion. However, till now, it is still unclear of the molecular regulatory network during auxin-mediated peach fruit expansion.

**Results:**

Here, exogenous NAA application markedly increased IAA content and drastically decreased ABA content at the fruit expansion stage. Correspondingly, NAA mainly induced the auxin biosynthesis gene (1 *PpYUCCA)* and early auxin-responsive genes (7*PpIAA*, 3 *PpGH3*, and 14 *PpSAUR)*; while NAA down-regulated ABA biosynthesis genes (2 *PpNCED*, 1 *PpABA3*, and 1 *PpAAO3*). In addition, many DEGs involved in other plant hormone biosynthesis and signal transduction were significantly enriched after NAA treatment, including 7 JA, 7 CTK, 6 ETH, and 3 GA. Furthermore, we also found that NAA treatment down-regulated most of genes involved in the growth and development of peach fruit, including the cell wall metabolism-related genes (*PpEG*), sucrose metabolism-related genes (*PpSPS*), phenylalanine metabolism-related genes (*PpPAL*, *Pp4CL*, and *PpHCT*), and transcription factors (*PpNAC*, *PpMADS-box*, *PpDof*, *PpSBP*, and *PpHB*).

**Conclusion:**

Overall, NAA treatment at the fruit expansion stage could inhibit some metabolism processes involved in the related genes in the growth and development of peach fruit by regulating multiple-hormone signaling networks. These results help reveal the short-term regulatory mechanism of auxin at the fruit expansion stage and provide new insights into the multi-hormone cascade regulatory network of fruit growth and development.

**Supplementary Information:**

The online version contains supplementary material available at 10.1186/s12870-023-04514-2.

## Background

Peach [*Prunus persica* (L.) Batsch] is generally classified as a climacteric fruit, popular among consumers because of its beautiful appearance, delicate flesh aromatic flavor, and high nutritional value [1]. Fruit expansion stage of peach is the key stage of fruit yield and quality formation, which is a complex and genetically programmed process that results in a whole set of physiological, biochemical, and structural changes in the color, flavor, aroma, texture, and nutritional value [2, 3].

Auxin is the first plant hormone to be discovered, which influences numerous facets of plant development and growth, notably cell division, elongation, and distinctions. crucial essential [4], apical dominance [5], root architecture development [6], the induction of blooming [7], and fruit development [8]. During flower development, auxin can serve as an alternative signal replacing pollination and fertilization to trigger fruit-setting [9]; during the young fruit stage, auxin promotes the expansion of fruit by promoting the elongation and enlargement of cells [10]; and during fruit ripening stage, auxin has more different and complex effects among different species, which promotes or inhibits fruit ripening and softening. In general, auxin is regarded as a negative regulator of fruit ripening. For example, in grape, auxin treatment delayed ripening onset and delayed harvest by inhibiting cell expansion [11]. In tomato, exogenous auxin delayed fruit ripening and strongly down-regulated the expression patterns of many genes related to carotenoid metabolism, cell degradation, and energy metabolism [12]. However, auxin promotes maturation by inducing ethylene release in some climacteric fruit. In peach, the release of ethylene increased, the firmness of fruit decreased, and the expression levels of *PpACSI*, *PpACO1*, *PpPG*, and *PpPME* were significantly increased after exogenous auxin treatment [13, 14]. Similarly, in apple, applying naphthaleneacetic acid (NAA) increased fruit ethylene production and stimulated the expression of *MdACS1*, *MdACO1*, and *MdERS1* genes [15]. In addition, different concentrations and treatment stages of auxin application also had different regulatory effects. In banana, treatment with 100 μM IAA delayed banana fruit ripening and inhibited ethylene production, but 57.1 μM IAA treatment showed the opposite result [16, 17]. In apple, NAA treatment failed to induce ethylene biosynthesis at 55 or 85 days after full bloom (DAFB), but strongly induced ethylene production at 115 DAFB [18]. In peach, auxin significantly induced ethylene production and promoted fruit softening and the induction of ethylene by auxin increased with treatment concentration at mature stage [13], however, at 30 days after full blossom, low concentrations of NAA (0.25 mM) promoted peach fruit maturity, while high concentrations of NAA (0.5 mM, 1 mM, 2 mM) inhibited fruit maturity and even caused fruit deformation and abscission [19]. Therefore, it is necessary to accurately verify the dosage and treatment period of auxin.

Plant hormones do not act in isolation in the process of plant growth, development, maturity, and senescence. On the contrary, many endogenous and exogenous plant hormones, such as auxin (AUX), ethylene (ETH), abscisic acid (ABA), and gibberellic acid (GA), operate together or against one another to govern plant growth and development [3]. In peach, *PpIAA1* and *PpERF4* form a positive feedback loop to regulate peach fruit ripening by integrating auxin and ethylene signals [14]. Similarly, in papaya, an auxin response factor *CpARF2* interacted with an important ethylene signal transcription factor *CpEIL1*, thus increasing the ripening-associated genes *CpACS1*, *CpACO1*, *CpXTH12*, and *CpPE51* [20]. Previous studies suggest that there is crosstalk between ABA and ethylene and that ABA regulates fruit ripening via ethylene signal. In climacteric fruits, ABA can affect ethylene production by regulating the expression of genes related to ethylene synthesis, such as *ACS* and *ACO*; while ethylene also regulates the expression of the ABA biosynthesis gene *PpNCED* through *PpERF*3 [21–23]. In addition, ABA, brassinosteroids, and jasmonic acid can also promote ripening in climacteric fruits, where they modulate the concentration of *ACO* and/or *ACS* transcripts to enhance ethylene levels [24]. Thus, exploring the crosstalk among plant hormones regulating fruit growth and development seems to be of great significance.

Although previous studies have shown that auxin plays significant roles in the growth and development of climacteric fruit through mediating multi-hormone signals. However, the molecular mechanisms and regulatory networks of auxin-induced fruit growth and development at the fruit expansion stage are still unclear. Therefore, auxin treatments were applied to peach at the fruit expansion stage. Firstly, HPLC-MS and GC-MS were used to determine the hormonal changes after auxin treatment, Then, transcriptomic analysis was used to develop a multi-hormone regulatory network in response to auxin, Finally, the differentially expressed genes (DEGs) analysis revealed the auxin-mediated regulation mode of sucrose metabolism, cell wall metabolism, phenylalanine metabolism, and transcription factors. These results provide new insights into the auxin-mediated multi-hormone regulatory network at peach fruit expansion stage.

## Results

### RNA-Seq and pathway enrichment analysis

#### Transcriptome analysis and biological pathways identification during peach fruit development

**Fig. 1:**
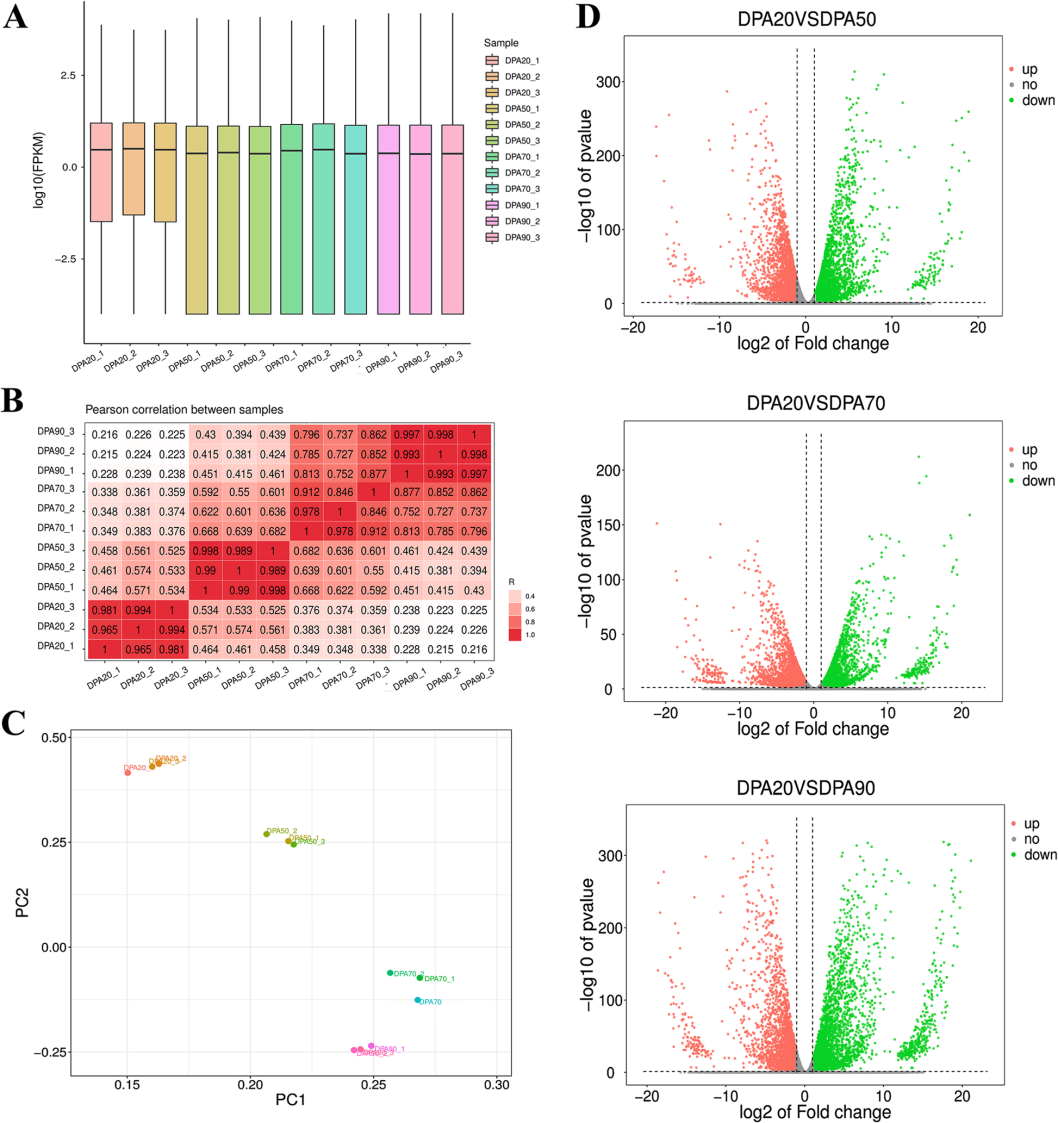
Comparison of transcriptomes of different stages of peach fruit development. **A** Boxplot of gene expression distribution. The abscissa is the sample name, and the ordinate is log10 (FPKM). **B** Pearson correlation between samples. **C** Principal component analysis of samples. Different colors indicate different developmental stages. **D** Volcano map of other compare groups. Genes with significant differential expression are indicated by red dots (up-regulated), green dots (down-regulated), and gray dots (no significant change)

The growth and development of peach fruit presented a typical double-sigmoidal curve, which included four stages, namely, the first exponential growth stage (20DPA), the pit hardening stage (50DPA), the second exponential growth stage (70DPA), and mature stage (90DPA). In this study, the peach flesh samples of four developmental stages were selected for dynamic transcriptome analysis, and each sample consisted of three independent replicates. After read filtering, 79.86 Gb clean reads were obtained from the 12 samples, with an average of 6.66 Gb clean reads per sample, of which 80.82%~87.03% clean reads were uniquely mapped to the peach reference genome (Supplementary Table S2). Moreover, fragments per kilobase of transcript per million mapped reads (FPKM) density distribution profiles were created to depict the gene expression profile of each sample (Fig. 1A). According to the results of Pearson correlation coefficient analysis and principal component analysis (Fig. 1B and C), we observed significant differences between the four stages and high reproducibility between the three biological replicates of the samples. These data demonstrated that the considerable quality of transcriptome sequencing could be used for further analysis.

**Fig. 2:**
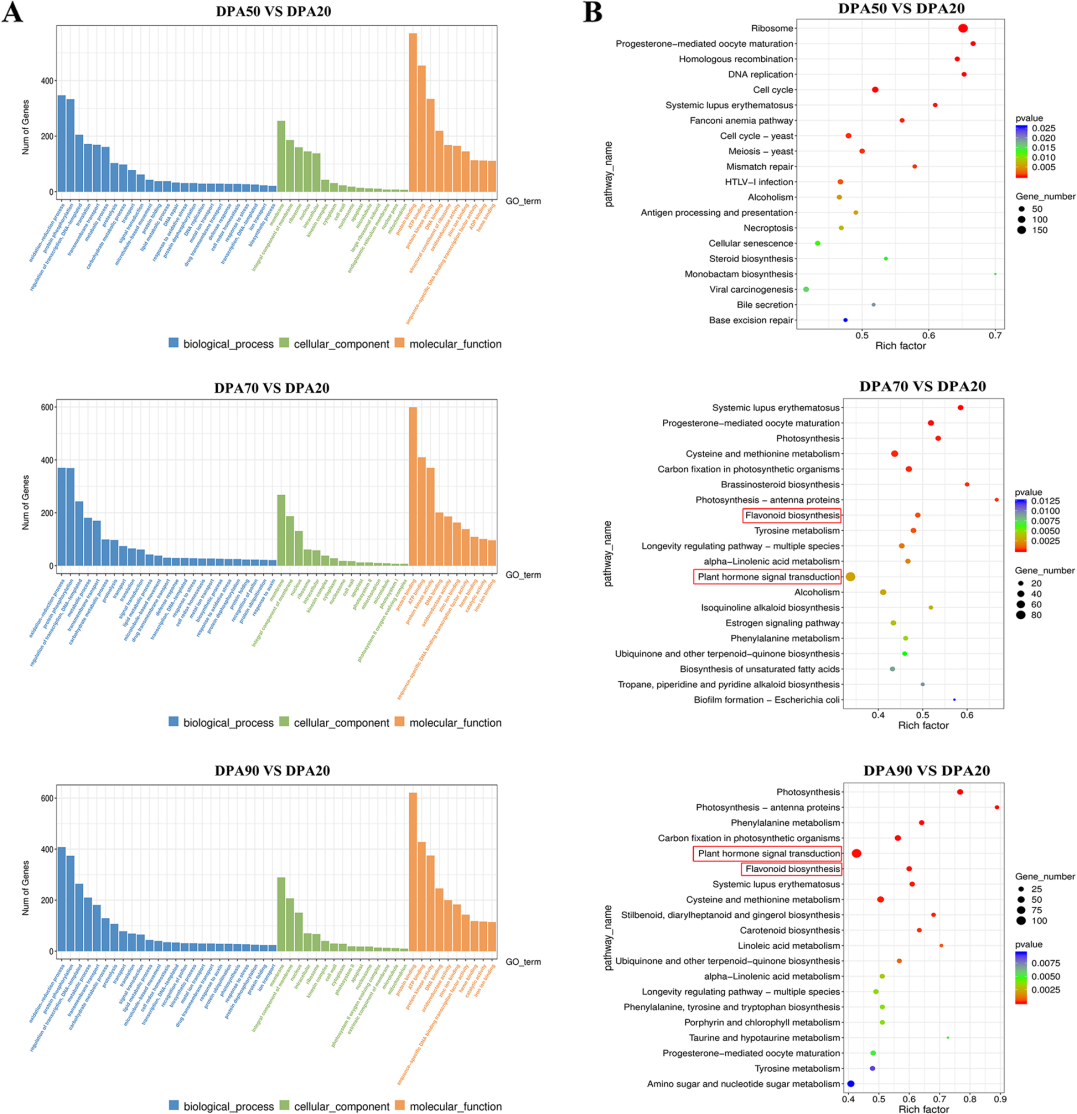
GO and KEGG enrichment analysis of the DEGs. **A** The GO terms of the top 50 significantly enriched pathways. The different colors are used to distinguish biological processes (BP), cellular components (CC), and molecular functions (MF). **B** The KEGG pathway terms of the top 20 significantly enriched pathways. The number of DEGs is demonstrated through the magnitude of the dot, and the color of the dot stipulates the severity of DEGs

In this study, three comparison groups (DPA20 vs. DPA50, DPA20 vs. DPA70, and DPA20 vs. DPA90) were selected, which produced 7032, 6885, and 7656 DEGs, respectively (Fig. 1D and Supplementary Table S3). Further, the DEGs were annotated using the GO and KEGG databases. In the DPA50 vs. DPA20 comparison group, 3893 DEGs were enriched to 913 GO terms, and 1654 DEGs were enriched to 347 KEGG pathway terms. In the DPA70 vs. DPA20 comparison group, 3746 DEGs were enriched to 853 GO terms, and 1349 DEGs were enriched to 341 KEGG pathway terms. In the DPA90 vs. DPA20 comparison group, 4180 DEGs were enriched to 928 GO terms, and 1601 DEGs were enriched to 351 KEGG pathway terms. Among these terms, the top 50 GO terms and the top 20 KEGG pathway terms were selected and displayed in Fig. 2A and 2B. Particularly, in the DPA70 vs. DPA20 and DPA90 vs. DPA20 comparison groups, the DEGs were significantly enriched in Plant hormone signal transduction (ko04075) and Flavonoid biosynthesis (ko00941). These results indicated that 70DPA might be a critical period for peach fruit to enter the ripening process, with significant changes in hormone levels and color during this period.

#### Transcriptome analysis and biological pathways identification in response to auxin treatment

**Fig. 3:**
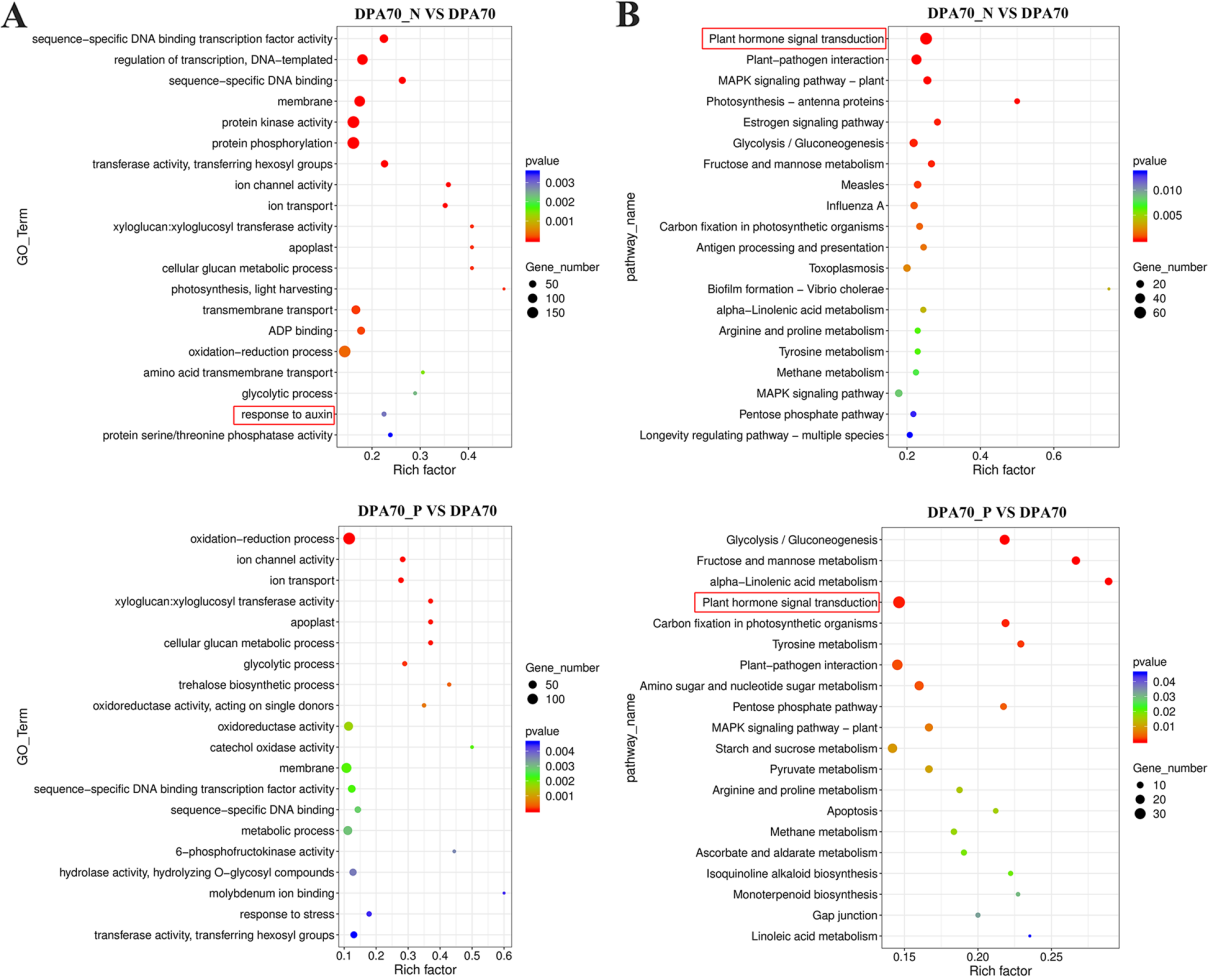
Enrichment analysis of the DEGs in the DPA70_N vs. DPA70 and DPA70_P vs. DPA70 comparison groups. **A** The scatter plot of GO enrichment of the top 20. **B** The scatter plot of KEGG enrichment of the top 20. The abscissas represent the Rich factor, and the ordinates represent the pathway terms. The size of the dot indicates the number of DEGs, and the color of the dot indicates the significance of DEGs

To examine the effect of auxin on peach fruit growth and development, transcriptome sequencing of peach fruit samples treated with NAA and NPA was performed. After removing reads containing adapter, reads containing Nbase, and low-quality reads, these two samples generated 5.86 and 6.92 Gb clean reads, respectively (Supplementary Table S2). After NAA treatment, 867 DEGs (30.60%) were up-regulated, 1966 DEGs (69.40%) were down-regulated; while after NPA treatment, 1139 DEGs (55.29%) were up-regulated, and 921 DEGs (44.71%) were down-regulated (Supplementary Table S3). GO databases were divided into three major functional categories: molecular function (MF), cellular component (CC), and biological process (BP). In the DPA70_N vs. DPA70 comparison group, a total of 1535 DEGs were enriched to 465 GO terms, including 168 BP, 40 CC, and 254 MF; in the DPA70_P vs. DPA70, a total of 1081 DEGs were enriched to 455 GO terms, including 163 BP, 45 CC, and 244 MF. Furthermore, the KEGG enrichment results showed that 520 DEGs were enriched to 316 KEGG pathways in the DPA70_N vs. DPA70 comparison group, and 430 DEGs were enriched to 295 KEGG pathways in the DPA70_P vs. DPA70 comparison group. Interestingly, 18 DEGs were significantly enriched in response to auxin (GO: 0009733) and 62 DEGs were significantly enriched in Plant hormone signal transformation (ko04075) in the DPA70_N vs. DPA70 comparison group; while 36 DEGs were significantly enriched in Plant hormone signal transformation (ko04075) in the DPA70_P vs. DPA70 comparison group (Fig. 3). The analysis implied that auxin can regulate the growth and development of peach fruit at the fruit expansion stage by mediating multi-hormone signaling.

### Effects of exogenous auxin on endogenous plant hormones

#### Expression analysis of auxin-related genes during peach fruit development

**Fig. 4:**
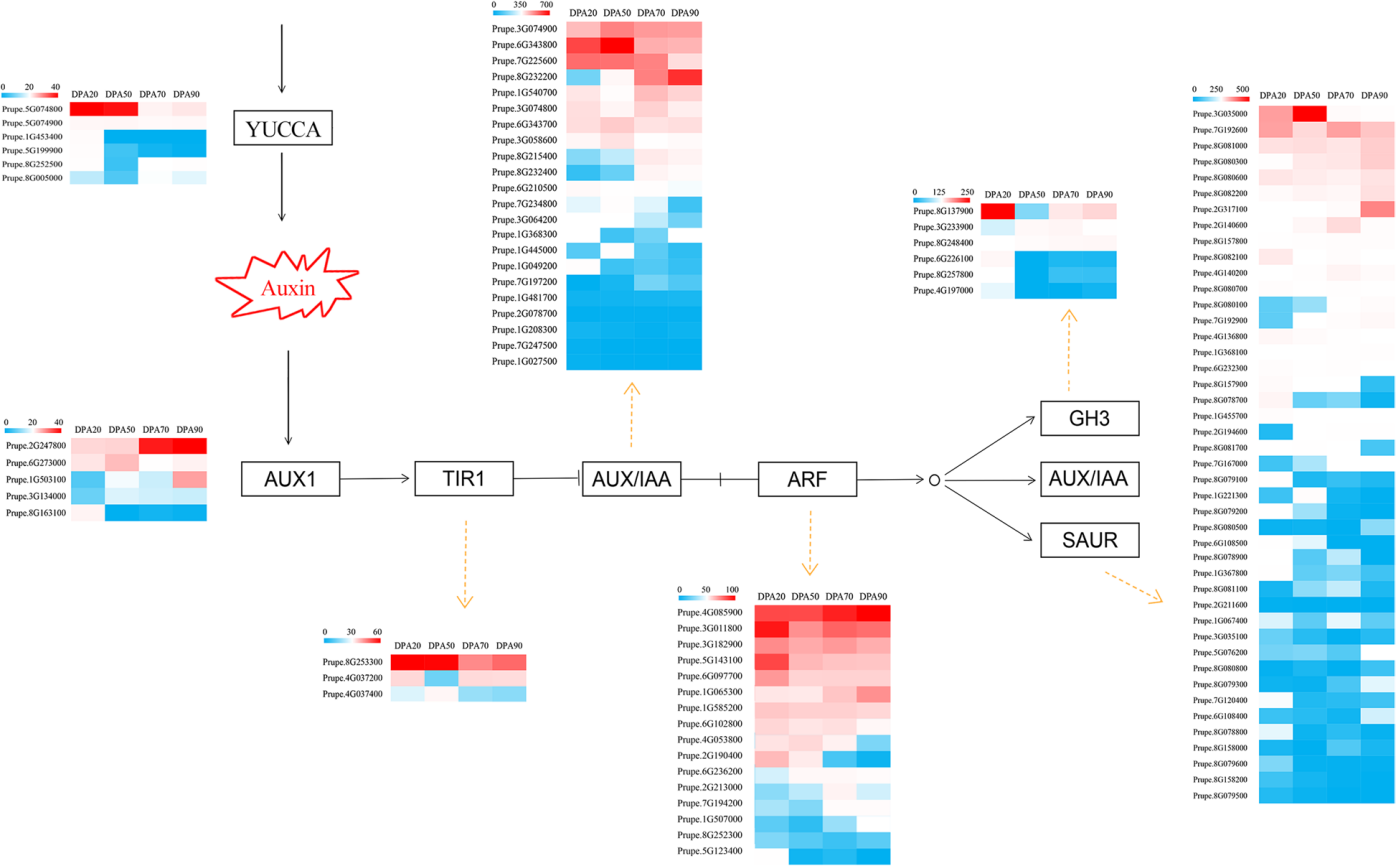
Expression heatmap of auxin biosynthesis and signal transduction genes during peach fruit development. Blue indicated low expression, and red indicated high expression

From transcriptome data, a total of 87 DEGs involved in auxin biosynthesis and signal transduction were identified, including 5 auxin influx carriers (AUX1), 3 transport inhibitor response 1 (TIR1), 18 auxin/indole-3-acetic acid (AUX/IAA), 15 auxin response factor (ARF), 6 Gretchen Hagen 3 (GH3), 34 small auxin-up RNA (SAUR), and 6 YUCCA (Supplementary Table S4 and Fig. 4). In the auxin biosynthesis and signaling transduction pathway, 17 DEGs (1 *PpAUX1*, 3 *PpARF*, 1 *PpIAA*, 4 *PpGH3*, 6 *PpSAUR*, and 2 *PpYUCCA*) were significantly highly expressed at 20DPA, 5 DEGs (2 *PpIAA*, and 3 *PpSAUR*) at 50DPA, 2 DEGs (1 *PpARF* and 1 *PpSAUR*) at 70DPA, and 3 DEGs (1 *PpAUX1* and 2 *PpSAUR*) at 90DPA, indicating that these auxin-related genes are involved in the specific stages of peach fruit growth and development. Moreover, 4 DEGs (2 *PpTIR1*, 1 *PpIAA*, and 1 *PpYUCCA*) were highly expressed at 20DPA and 50DPA, which mainly played roles in the early stage of peach fruit development. In comparison, 5 DEGs (1 *PpARF*, 3 *PpIAA*, and 2 *PpSAUR*) were highly expressed at 70DPA and 90DAPA, which mainly played roles in the late stage of peach fruit development. With the growth and development of the peach fruit, the expression of 11 genes (3 *PpARF*, 1 *PpIAA*, 1 *PpGH3*, and 6 *PpSAUR*) showed an upward trend, which might promote fruit ripening; while the expression of 7 genes (2 *PpARF*, 3 *PpIAA*, and 2 *PpSAUR*) showed a downward trend, which might inhibit fruit ripening.

#### Mult-hormone biosynthesis and signaling genes in response to auxin treatment

The growth and development of peach fruits are co-regulated by multiple hormones. In order to understand the response of multiple hormones to auxin during the peach fruit expansion period, six plant hormones were identified in this exploration, including 3-indoleacetic acid (IAA), abscisic acid (ABA), methyl jasmonate (MeJA), ethylene (ETH), gibberellin (GA), and trans-zeatin (ZT). Among them, ethylene (ETH) is not produced at the fruit expansion stage. Compared with the control group, NAA treatment significantly increased the content of IAA and reduced the content of ABA; While NPA treatment significantly reduced the content of IAA and ZT (Supplementary Fig. S1).

**Fig. 5:**
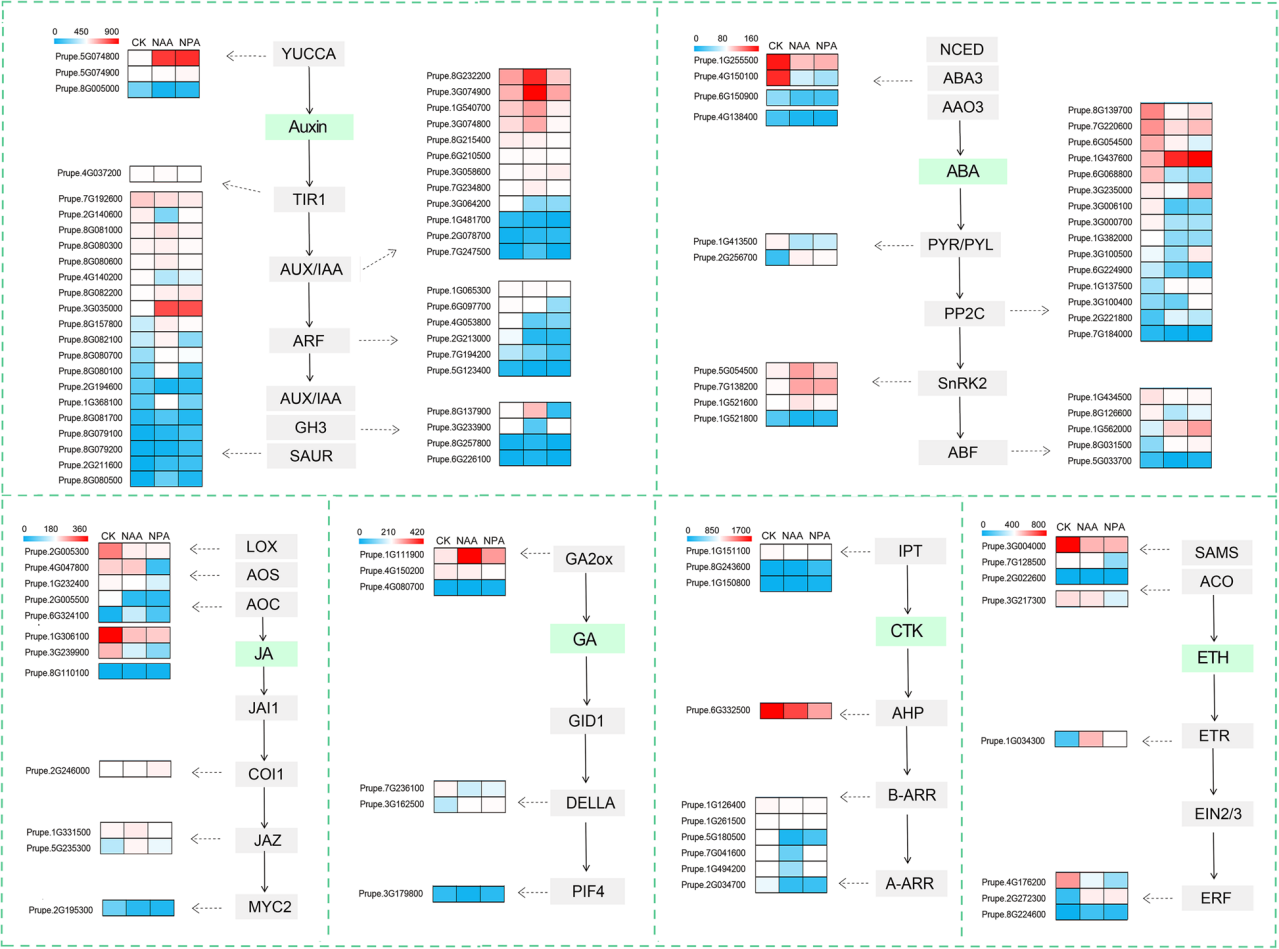
Expression heatmap of six plant hormone biosynthesis and signaling genes in response to auxin treatment. Blue indicated low expression, and red indicated high expression

Furthermore, the DEGs of six plant hormone biosynthesis and signal transduction were identified (Fig. 5). After NAA treatment, a total of 89 DEGs of the plant hormone biosynthesis and signal transduction were significantly enriched, including 39 AUX, 27 ABA, 7 JA, 7 CTK, 6 ETH, and 3 GA (Supplementary Table S5). And after NPA treatment, a total of 68 DEGs of the plant hormone biosynthesis and signal transduction were significantly enriched, including 25 AUX, 17 ABA, 9 JA, 8 CTK, 6 ETH, and 3 GA (Supplementary Table S6). In the auxin biosynthesis pathway, 1 *PpYUCCA* (Prupe.5G074800) exhibited significantly up-regulated by NAA treatment. Meanwhile, 24 early auxin-responsive genes, such as 7 *PpIAA*, 3 *PpGH3*, and 14 *PpSAUR*, were rapidly induced and up-regulated by NAA treatment. In addition, 4 *PpARF* and 3 *PpAUX1* were down-regulated by NAA treatment. Moreover, NAA treatment significantly affected the expression abundance of genes involved in ABA biosynthesis and signaling pathways. As the rate-limiting enzymes for ABA biosynthesis, 2 *PpNCED*, 1 *PpABA3*, and 1 *PpAAO3* were down-regulated by NAA treatment. And in ABA signaling pathways, 14 DEGs (1 *PpPYL*, 10 *PpPP2C*, 1 *PpSnRK2*, and 2 *PpABF*) were down-regulated, while 9 DEGs (1 *PpPYL*, 4 *PpPP2C*, 2 *PpSnRK2*, and 2 *PpABF*) were up-regulated. In the ethylene biosynthesis and signal transduction pathway, 6 DEGs were regulated by NAA treatment. Among them, 2 *PpSAMS* (Prupe.2G022600, Prupe.3G004000) were strongly down-regulated, and 1 *PpETR* (Prupe.1G034300) was significantly induced. Furthermore, 2 *PpERF* (Prupe.2G272300, Prupe.8G224600) were up-regulated, and 1 *PpERF* (Prupe.4G176200) was down-regulated, indicating that these *PpERF* genes might play different roles in response to auxin treatment during peach development. In the JA biosynthetic pathway, the expression of *PpAOC* (Prupe.1G306100, Prupe.3G239900) and *PpLOX2* (Prupe.2G005300, Prupe.2G005500) decreased significantly in response to NAA treatment. And in the JA signal transduction pathway, 1 *PpJAZ* gene was up-regulated, and 1 *PpMYC2* gene was significantly down-regulated. As a critical enzyme in the gibberellin biosynthesis pathway, GA2ox can inhibit GA synthesis. In this work, *PpGA2ox* (Prupe.1G111900) was significantly up-regulated after NAA treatment; on the contrary, *PpDELLA* (Prupe.7G236100) and *PpPIF4* (Prupe.3G179800) were significantly down-regulated. Interestingly, all DEGs (1 IPT, 1 B-ARR, and 5 A-ARR) were significantly down-regulated in the cytokinin biosynthesis and signal transduction pathway.

#### Effects of exogenous auxin on the peach fruit development at the fruit expansion stage

### Cell wall metabolism in response to auxin treatment

The fruit cell wall is a complex structure composed of pectin, cellulose, hemicellulose, and glycoprotein. The change in cell wall structure and composition is essential for fruit ripening and softening. In this study, 13 DEGs involved in cell wall metabolism were enriched after auxin treatment (Supplementary Table S7), including Endo-1,4-β-D-glucanase (EG), pectin esterase (PE), pectate lyases (PL), and xyloglucan:xyloglucosyl transferase (XET/XTH). After NAA treatment, 10 DEGs involved in cell wall metabolism were enriched, 3 DEGs (1 *PpPE*, 1 *PpPL*, and 1 *PpXTH*) were increased, and 7 DEGs (4 *PpEG*, 1 *PpPE*, 1 *PpPL*, and 1 *PpXTH*) were significantly reduced. And after NAP treatment, a total of 12 DEGs involved in cell wall metabolism were enriched, 5 DEGs (1 *PpEG*, 2 *PpPE*, and 2 *PpXTH*) were up-regulated, and 7 DEGs (3 *PpEG*, 3 *PpPL*, and 1 *PpPE*) were significantly down-regulated. Notably, 1 DEG (*PpXTH*) was significantly up-regulated, and 5 DEGs (3 *PpEG*, 1 *PpPL*, and 1 *PpPE*) were significantly down-regulated after NAA and NPA treatment.

### Sucrose metabolism in response to auxin treatment

The soluble sugars in peach fruit are mainly sucrose, glucose, fructose, and sorbitol, and the sucrose content is the highest. Sucrose phosphate synthase (SPS) is a pivotal enzyme that catalyzes sucrose formation, which converts UDP: uridine diphosphate glucose and fructose-6-phosphate into sucrose-6-phosphate and other converts sucrose-6-phosphate into sucrose-by-sucrose phosphate phosphatase (SPP). Sucrose synthase (SUS), a soluble enzyme in the cytoplasm, has two isoforms, SS I have the activity of sucrose degradation, and SS II has the activity of sucrose biosynthesis. Invertase (INV) can irreversibly catalyze the hydrolysis of sucrose to glucose and fructose. In addition, fructokinase (FK) and hexokinase (HK) play a significant role in sucrose metabolism, which can catalyze the formation of glucose-6-phosphate and fructose-6-phosphate from glucose and fructose. In this study, 9 DEGs of the sucrose metabolism pathway were identified (Fig. S2 and Supplementary Table S8), including 3 *PpSPS*, 3 *PpSUS*, 1 *PpINV*, 1 *PpFK*, and 1 *PpHK*. Interestingly, 3 *PpSUS*, 1 *PpFK*, 1 *PpHK*, and 1 *PpINV* were significantly up-regulated and 1 *PpSPS* was significantly down-regulated after NAA and NPA treatments.

### Phenylalanine metabolism in response to auxin treatment

The phenylpropanoid pathway is an essential pathway for synthesizing secondary plant metabolites, which includes phenylalanine metabolism and the downstream branching of secondary metabolites, such as synthesizing flavonoids, lignin, and chlorogenic acid. In this study, 8 DEGs of the phenylpropanoid pathway were identified, including phenylalanine ammonia-lyase (PAL), cytochrome P450 98A (CYP98A, C3H), cytochrome P450 75B1 (CYP75B1, F3’H), 4-coumarate:CoA ligase (4CL), hydroxycinnamoyl-CoA shikimate/quinate hydroxycinnamoyl transferase (HCT), leucoanthocyanidin dioxygenase (LDOX), caffeoyl-CoA O-methyltransferase (CCoAMT) (Supplementary Table S9). After NAA treatment, 1 DEG (*PpC3H*) was significantly up-regulated, and 3 DEGs (*PpPAL*, *Pp4CL*, and *PpHCT*) were significantly down-regulated. After NPA treatment, 3 DEGs (*PpC3H*, *PpHCT*, and *PpLDOX*) were significantly up-regulated, and 2 DEG (*PpCCoAMT* and *PpF3’H*) was significantly down-regulated. Notably, *PpC3H* (Prupe.1G580500) is up-regulated after NAA and NPA treatments.

### Transcription factors in response to auxin treatment

**Fig. 6:**
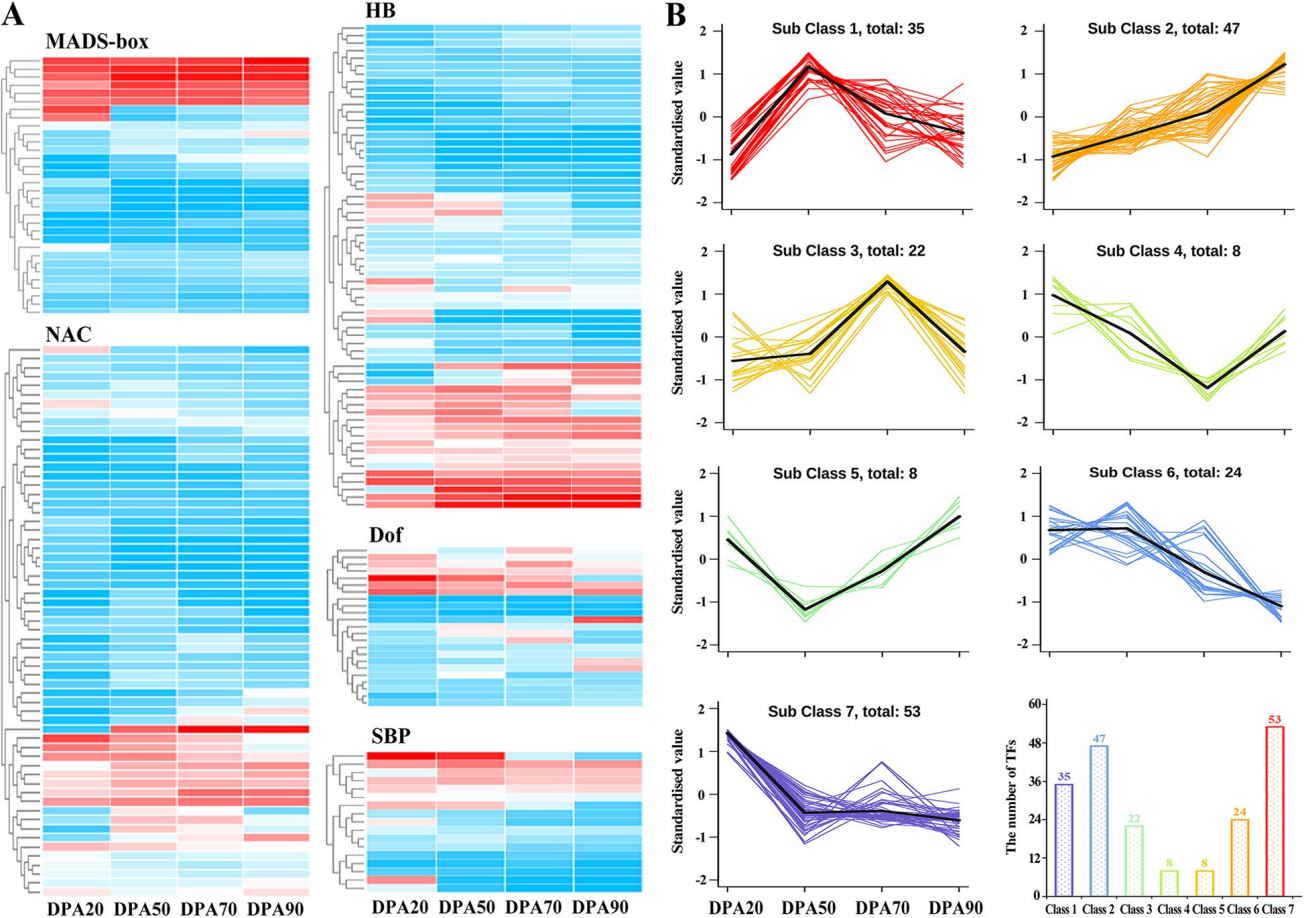
Screening of TFs during peach fruit development. **A** Gene expression heatmap of 197 TF. The color ranges from red to blue, indicating log_10_(FPKM) from large to small. **B** K-means cluster analysis of 197 TFs. All data are normalized using the z-score

Transcription factors (TFs) are essential in regulating gene expression in plants. TFs can bind to various specific DNA elements upstream of the target gene and activate or inhibit the transcriptional activity of the target gene from regulating its spatial-specific expression [1]. In addition to hormone-related transcription factors (Figs. 4 and 5), other transcription factors such as NAC, MADS-box, Dof, SBP, and HB have also been reported to be involved in the growth and development of peach fruit. 197 TFs were identified in this study, including 63 HB, 61 NAC, 33 MADS-box, 23 Dof, and 17 SBP (Fig. 6A). By K-means cluster analysis, 7 model expression profiles were obtained. With the growth and development of peach fruit, Sub Class 2 (47 TFs) showed an upward trend, which might promote peach fruit ripening; Sub Class 6 (24 TFs) and Sub Class 7 (53 TFs) showed a downward trend, which might inhibit peach fruit ripening; and Sub Class 4 (8 TFs) and Sub Class 5 (8 TFs) showed a changing trend of falling first and then rising. In addition, Sub Class 1 (35 TFs) showed significantly high expression at 50DAF, indicating that these TFs might regulate the growth and development of peach fruit at the pit hardening stage. Sub Class 3 (22 TFs) showed significantly high expression at 70DAF, indicating that these TFs might regulate the growth and development of peach fruit at the second exponential growth stage (Fig. 6B). After NAA treatment, 50 DEGs were significantly enriched, including 11 up-regulated TFs (2 Dof, 3 HB, 2 MADS-box, and 4 NAC) and 39 down-regulated TFs (4 Dof, 11 HB, 4 MADS-box, 16 NAC, and 4 SBP). And after NPA treatment, 33 DEGs were significantly enriched, including 19 up-regulated TFs (2 Dof, 2 HB, 5 MADS-box, 9 NAC, and 1 SBP) and 14 down-regulated TFs (2 Dof, 4 HB, 7 NAC, and 1 SBP (Supplementary Fig. S3 and Supplementary Table S10).

### Auxin regulates the growth and development of peach fruit at the expansion stage by mediating multiple-hormone signaling

**Fig. 7:**
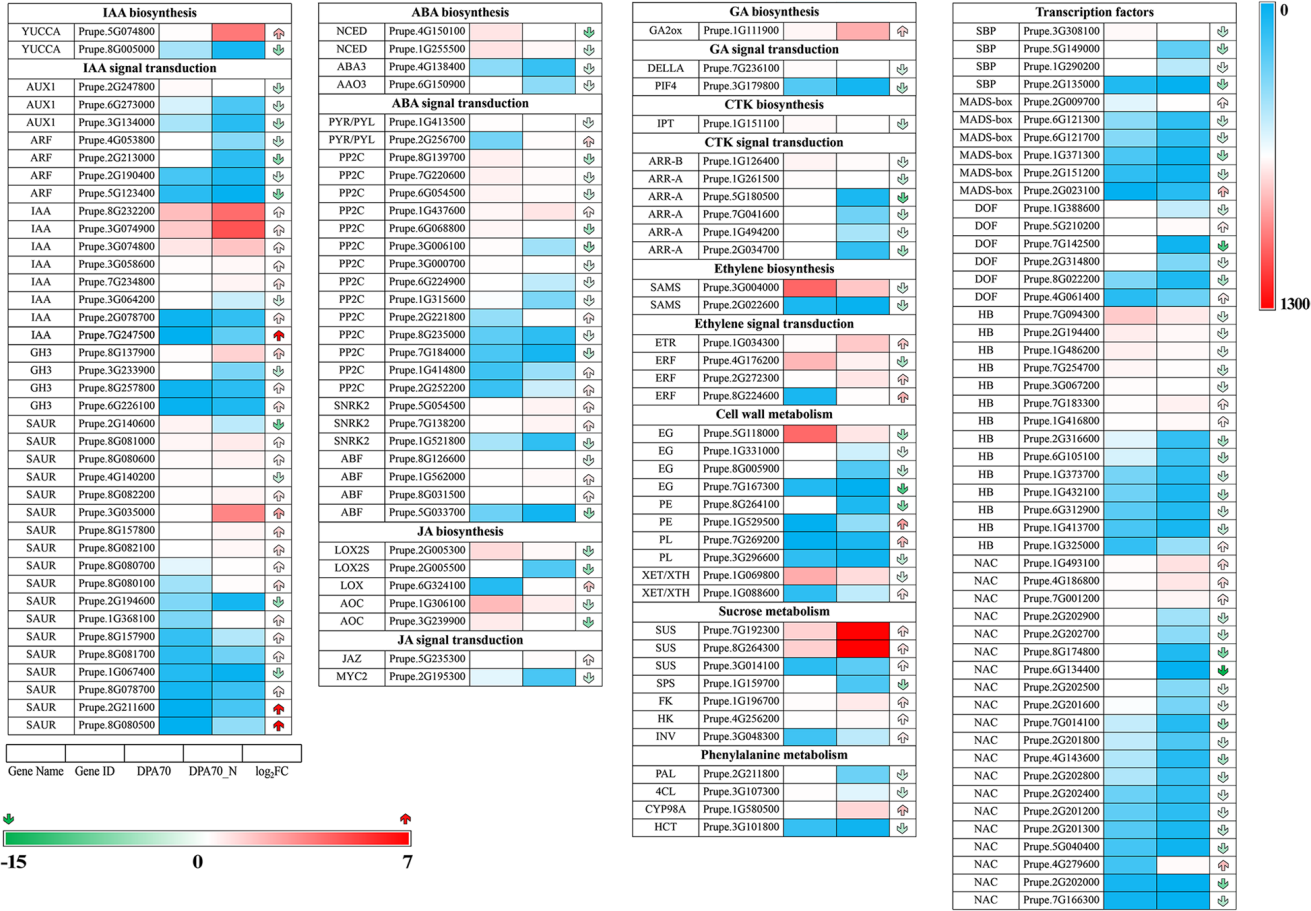
Expression heatmap of 160 DEGs involved in the plant hormone biosynthesis and signaling transduction, sucrose metabolism, cell wall metabolism, phenylalanine metabolism, and transcription factors in response to auxin treatment. Blue indicated low expression, and red indicated high expression

In this study, we identified 160 DEGs after NAA treatment, of which 89 DEGs were involved in the plant hormone biosynthesis and signal transduction, 10 DEGs were involved in the sucrose metabolism, 7 DEGs were involved in the cell wall metabolism, 4 DEGs were involved in the phenylalanine metabolism, and 50 DEGs were involved in the transcription factors (Fig. 7). After NAA treatment, 40 DEGs involved in the plant hormone biosynthesis and signal transduction were significantly up-regulated, 5 IAA, 9 ABA, 2 JA, 3 ETH, and 1 GA, which were positively correlated with the changes in the IAA content; while 49 DEGs involved in the plant hormone biosynthesis and signal transduction were significantly down-regulated, 14 AUX, 18 ABA, 5 JA, 7 CTK, 3 ETH, and 2 GA, which were negatively correlated with the changes in the IAA content. Interestingly, NAA significantly up-regulated most of the auxin biosynthesis and signal transduction genes and down-regulated most of the auxin biosynthesis and signal transduction genes, which is consistent with the changes in the IAA and ABA content after NAA treatment. In addition, NAA treatment down-regulated most of genes involved in the growth and development of peach fruit, the cell wall metabolism-related genes (*PpEG*), sucrose metabolism-related genes (*PpSPS*), phenylalanine metabolism-related genes (*PpPAL*, *Pp4CL*, and *PpHCT*), and transcription factors (*PpNAC*, *PpMADS-box*, *PpDof*, *PpSBP*, and *PpHB*), which were negatively correlated with the changes in the IAA content. Therefore, we speculated that NAA treatment at the fruit expansion stage could inhibit the growth and development of peach fruit by regulating multiple-hormone signaling networks.

### qRT-PCR validation of DEGs from RNA-seq

**Fig. 8:**
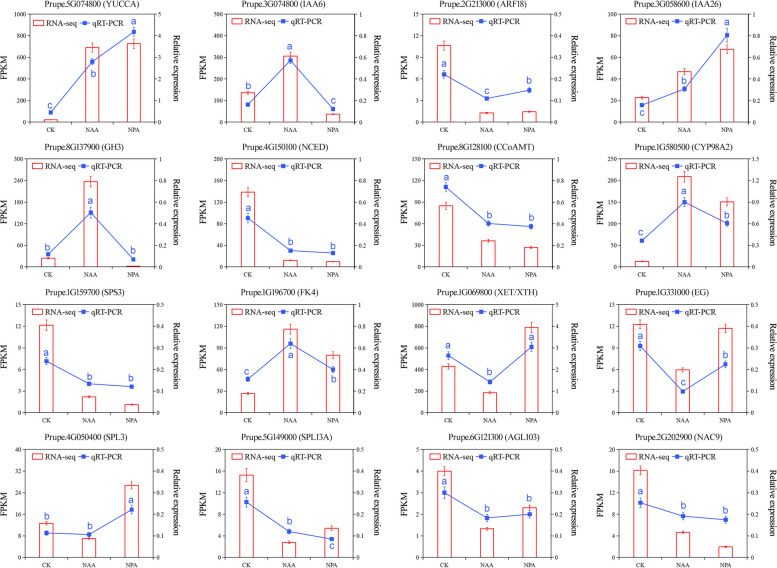
Validation of RNA-seq by qRT-PCR. Comparisons between different treatments were performed by a one-way analysis of variance (ANOVA) test at a significance level of P<0.05. Different lowercase letters indicate a significant difference at the 5% level between different treatments

To confirm the accuracy and reproducibility of the expression profiles obtained from the RNA-seq data, 16 DEGs were chosen randomly for qRT-PCR analysis after NAA and NPA treatments (Supplementary Table S1 and Fig. 8). Those genes were involved in auxin biosynthesis and signal transduction (*PpYUCCA*, *PpIAA*6, *PpIAA*26, *PpARF*18, and *PpGH3*), ABA biosynthesis and signal transduction (*PpNCED*), phenylalanine metabolism (*PpCCoAMT*, *PpCYP98A2*), sucrose metabolism (*PpSPS*3, *PpFK*4), cell wall metabolism (*PpXTH*, *PpEG*), and transcription factor (PpSPL3, PpSPL12A, PpAGL103, and *PpNAC*9). For the 16 candidate genes tested, the results of qRT-PCR agreed with the changes in transcript abundance expression profiles by RNA-seq, indicating the accuracy and reliability of the RNA-seq data.

## Discussion

### Identification and expression analysis of auxin-related genes

Auxin is an important Plant hormone, which plays an important regulatory role in all stages of plant growth and development. In this study, a total of 87 DEGs involved in auxin biosynthesis and signal transduction were identified from transcriptome data, including 6 DEGS (6 *PpYUCCA*) related to auxin biosynthesis and 81 DEGs (5 *PpAUX1*, 3 *PpTIR1*, 18 *PpIAA*, 15 *PpARF*, 6 *PpGH3*, and 34 *PpSAUR*) related to auxin signal transduction. YUCCA is a rate-limiting enzyme gene involved in the auxin biosynthesis pathway, and overexpression of the YUCCA gene promoted auxin synthesis in potato [25]. In this study, the expression levels of *PpYUCCA* varied at different developmental stages of peach fruit, 1 *PpYUCCA* was highly expressed at the young fruit stage, 2 *PpYUCCA* at the young fruit and pit hardening stage, 2 at the young fruit, expansion, and mature stage, which might participate in the synthesis of auxin at different periods. ARF and Aux/IAA are important proteins in the auxin signaling transduction pathway [26]. In this study, 3 *PpARF* and 1 *PpIAA* were highly expressed at the young fruit stage, 2 *PpIAA* at the pit hardening stage, 1 *PpIAA* at the young fruit and pit hardening stage, 1 *PpARF* at the expansion stage, 1 *PpARF* and 3 *PpIAA*at the expansion, and mature stage, indicating that these auxin-related genes are involved in the specific stages of peach fruit growth and development. Moreover, with the growth and development of the peach fruit, 3 *PpARF* and 1 *PpIAA* showed an upward trend, which might promote fruit ripening; while 2 *PpARF* and 3 *PpIAA* showed a downward trend, which might inhibit fruit ripening. Overall, these results indicated that members of the *PpARF* and *PpIAA* families had their specific functions during plant growth and development, but also shared redundant functions with other members of the family [27].

### Mult-hormone in response to auxin at the fruit expansion stage

Peach fruit growth and development is a complex process controlled and integrated by multiple hormone signals, with some hormones acting as activators and others as inhibitors [2]. Numerous studies have shown that auxin regulated fruit growth and development through interaction with other hormones [28]. In this study, NAA treatment significantly increased the content of IAA and reduced the content of ABA; While NPA treatment significantly reduced the content of IAA and ZT. It's worth noting that ethylene was not produced at the fruit expansion stage after NAA treatment.

Auxin has an important regulatory role in the growth and development of peach fruit. After NAA treatment, an auxin synthesis gene *PpYUCCA* (Prupe.5G074800) increased 32 times, indicating that NAA treatment stimulated the accumulation of auxin by inducing the expression level of *PpYUCCA* genes. Meanwhile, 24 early auxin-responsive gene families, including 7*PpIAA*, 3 *PpGH3*, and 14 *PpSAUR*, were rapidly induced and up-regulated by NAA treatment, consistent with the previous conclusions [29]. In addition, 4 *PpARF* and 3 *PpAUX1* were down-regulated in response to NAA treatment, indicating a negative feedback regulation of NAA biosynthesis.

ABA is a potent accumulation hormone in mature fruits, essential in regulating the ripening process in climacteric and non-climacteric fruits [30]. With the fruit ripening of fruit, the expression of ABA biosynthesis and signal-related genes increased, and *PpNCED*1 and *PpNCED*2 promoted ripening and senescent by regulating ABA synthesis [31]. In addition, ABA can promote fruit ripening by increasing the expression levels of ethylene synthesis genes (*PpACO1* and *PpACS1*) and auxin signaling genes [32]. In this study, NAA treatment significantly affected the gene expression abundance in ABA biosynthesis and signaling pathways. As the rate-limiting enzymes for ABA biosynthesis, 2 *PpNCED*, 1 *PpABA3*, and 1 *PpAAO3* were down-regulated by NAA treatment, which inhibited the ABA accumulation. And in the ABA signal transduction pathway, 14 DEGs were down-regulated and 9 DEGs were up-regulated, indicating that these genes in the ABA signal transduction pathway had different responses to NAA, but most of them were inhibited.

Ethylene is usually recognized as a trigger of the ripening process in climacteric fruits [33, 34]. Two systems influence the creation of ethylene in climacteric fruit: system 1 is only in charge of producing low basal ethylene quantities at the pre-climacteric stage, and system 2 is in control of producing a large amount of ethylene at the climacteric stage and controlling the ripening process through self-catalysis. Previous studies have found that a high concentration of auxin can stimulate the synthesis of system 2 ethylene through its inductive action on the expression of *PpACS1* in MF peaches during post-ripening; in contrast, the low concentrations of auxin due to the low expression of *PpYUC11* suppress *PpACS1* expression and ethylene production in SH peaches during post-ripening [13, 35, 36]. In this study, we found that ethylene was not produced at the fruit expansion stage after NAA treatment. In the ethylene biosynthesis pathway, two *PpSAMS* (Prupe.2G022600, Prupe.3G004000) were down-regulated; and in ethylene signal transduction pathway, two crucial rate-limiting enzymes, *PpACS* and *PpACO* did not change significantly. Therefore, we speculated that ethylene was not the primary regulatory hormone of peach fruit at the early fruit growth stage, and the dual effect of NAA on peach fruit ripening might be related to the changes in ethylene content.

In addition, we found that NAA treatment also promoted GA biosynthesis-related genes but inhibited JA and CTK biosynthetic-related genes. N-1-naphthylphthalamic acid (NPA) is an auxin polar transport inhibitor, and 68 DEGs of the plant hormone biosynthesis and signal transduction were significantly enriched after NPA treatment, including 25 AUX, 17 ABA, 9 JA, 8 CTK, 6 ETH, and 3 GA. However, we found that the regulation pattern of NPA was not completely antagonistic with that of auxin, and the specific molecular mechanism remains to be further studied. In conclusion, auxin application mainly triggered IAA biosynthesis and inhibited ABA biosynthesis of peach at the fruit expansion stage, suggesting a complex involvement of auxin and its interaction with phytohormones during the growth and development of peach fruit.

### Auxin inhibits some metabolism processes in fruit development by regulating cell wall, sucrose, and phenylpropanoid metabolisms

Fruit ripening and softening are accompanied by the degradation of pectin, cellulose, hemicellulose, and other cell wall substances, resulting from the interaction of various cell wall structure enzymes [37, 38]. In this process, polygalacturonase (PG), pectin esterase (PE), β-galactosidase (β-GAL), and pectate lyases (PL) are involved in the depolymerization of pectin; xyloglucan:xyloglucosyl transferase (XET/XTH), and Endo-1,4-β-D-glucanase (EG) are involved in the depolymerization of cellulose or hemicellulose. Polygalacturonase (PG) is an important enzyme involved in pectin degradation, which can be divided into endo-Polygalacturonase (Endo-PG) and exo-Polygalacturonase (Exo-PG). Studies have found that Endo-PG is a candidate gene for controlling the softening of peach fruit [39]. However, in this study, NAA treatment did not significantly affect *PpPG* genes at the fruit expansion stage, which may be due to the specific expression of *PpPG* genes during fruit ripening and softening. Endo-1,4-β-D-glucanase (EG) is a key enzyme that catalyzes the hydrolysis of cellulose, and the enzyme activity of EG is closely related to the fruit ripening of white pear [40]. Interestingly, the expression levels of 4 *PpEG* were significantly reduced after NAA treatment. Furthermore, the *PpPE*, *PpPL*, and *PpXTH* genes showed negative and positive regulation by NAA treatment, suggesting that these genes reflected diverse regulatory mechanisms in response to auxin. All in all, these findings indicated that NAA treatment inhibited the degradation of pectin and cellulose by directly controlling the expression of these softening-related genes.

Sucrose, glucose, fructose, and sorbitol are the primary sugar in peach fruit, which are not only essential to fleshy fruit growth and development but also central to fruit quality [41]. Sucrose phosphate synthase (SPS) is the key rate-limiting enzyme in regulating sucrose synthesis. In this study, *PpSPS1*, *PpSPS2*, and *PpSPS*3 showed an upward trend with the growth and development of peach fruit. After NAA treatment, *PpSPS*3 were significantly down-regulated, suggesting that NAA treatment inhibited sucrose synthesis. Sucrose synthesis (SUS) is a multigene family, and 6 *PpSUS* genes were identified in peach [42], 5 *VvSUS* genes in grape [43], 11 *MdSUS* genes in apple [44], and 30 *PbSUS* genes in pear [45]. SUS hasdual activity, SS I have the activity of sucrose degradation, and SS II has the activity of sucrose biosynthesis. In our study, different members of the *PpSUS* family had certain temporal expression specificity, and 3 *PpSUS* were significantly up-regulated after NAA treatment, which might exercise different functions in sucrose metabolism. In addition, invertase (INV) can irreversibly catalyze the hydrolysis of sucrose to glucose and fructose; and fructokinase (FK) and hexokinase (HK) can catalyze glucose and fructose to glucose-6-phosphate and fructose-6-phosphate [46]. In this study, NAA treatment up-regulated the expression of 1 *PpFK*, 1 *PpHK*, and 1 *PpINV*. Similarly, previous studies showed that sucrose accumulation in peach fruit involved the coordinated interaction of genes related to sucrose cleavage, resynthesis, and transport [47].

The phenylpropanoid metabolism pathway is essential for synthesizing plant secondary metabolites, including phenylalanine metabolism in the upstream and lignin, chlorogenic acid, and flavonoid biosynthesis in the downstream [48]. Phenylalanine ammonia-lyase (PAL), cinnamate-4-hydroxylase (C4H), and 4-coumarate:CoA ligase (4CL) are key genes of the upstream phenylpropane metabolic pathway, participate in the biosynthesis of multiple precursor compounds [49]. In the lignin synthesis pathway, the specificity of lignin is also regulated by ferulate 5-hydroxylase (F5H), caffeinated-5-O-methyltransferase (COMT), and caffeoyl-CoA 5-O-methyltransferase (CCoAOMT) [50]. And in the anthocyanin synthesis pathway, chalone synthase (CHS), chalone isomerase (CHI), flavonoid 3-hydroxylase (F3H), flavonoid 3'-hydroxylase (F3'H), dihydroflavonol reductase (DFR) to form leucoanthocyanidins, and then by leucoanthocyanin dioxygenase (LDOX) and UDP-glucose:flavonoid 3-O-glucosyltransferase (UFGT) to form various stable anthocyanidins [51]. Notably, *PpC3H* (Prupe.1G580500) was up-regulated after NAA and NPA treatments, indicating that *PpC3H* was responsive to auxin. In addition, 3 DEGs (*PpPAL*, *Pp4CL*, and *PpHCT*) were significantly down-regulated after NAA treatment. Overall, NAA treatment inhibited phenylpropanoid metabolism.

The growth and development of fruit are regulated by many transcription factors, which can be divided into positive and negative regulatory factors [52]. In tomato, NAC transcription factor NOR-like1 regulated genes involved in ethylene synthesis, chlorophyll degradation, carotenoid accumulation, and fruit softening and promote fruit ripening [53]. In papaya, a MADX-box *CpAGL18* activated the promoters of *CpACS1* and *CpSAUR32* and regulated fruit ripening via ethylene-auxin crosstalk [54]. In banana, Dof transcription factor *MaDof23* inhibited fruit ripening, while tomato transcription factor SlDof1 promoted fruit softening [55]. In this study, 197 TFs were identified, including 63 HB, 61 NAC, 33 MADS-box, 23 Dof, and 17 SBP, which regulated different stages of fruit development. For instance, 47 TFs in Sub Class 2 showed an upward trend, which might be positive regulatory factors of fruit growth;77 TFs in Sub Class 6 and Class 7 showed a downward trend, which might be negative regulatory factors of fruit growth. In addition, 39 TFs (4 Dof, 11 HB, 4 MADS-box, 16 NAC, and 4 SBP) were down-regulated, and only 11 TFs (2 Dof, 3 HB, 2 MADS-box, and 4 NAC) were up-regulated after NAA treatment, indicating that NAA mainly inhibits fruit ripening.

### Auxin-mediated multi-hormone to inhibit some metabolism processes in the development of peach fruit at the expansion stage

**Fig. 9:**
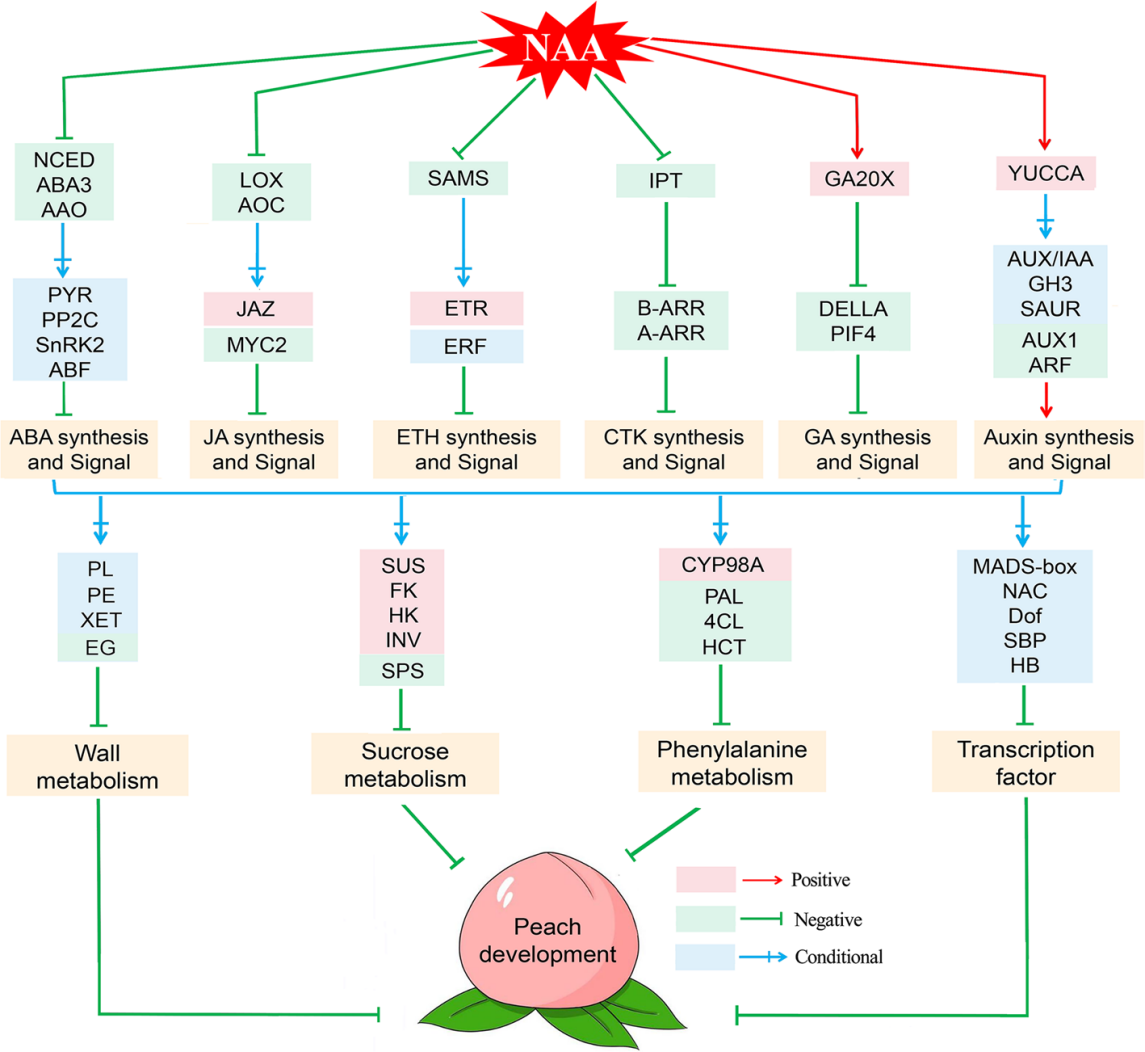
The putative molecular network of auxin regulates the development of peach by mediating multiple-hormone signaling at the fruit expansion stage

Peach [*Prunus persica* (L.) Batsch] is a typical representative of climacteric fruit, the growth and development of fruit is controlled and integrated by multiple hormone signals. Some studies have verified that auxin played significant roles in the ripening and softening of climacteric fruit through mediating multi-hormone signals. For example, the application of auxin at the later stage of fruit development can promote peach fruit ripening and softening by inducing ethylene production [13, 36]. However, the molecular mechanisms and regulatory networks of auxin-induced fruit growth and development at the fruit expansion stage are still unclear. Our work displays the important role of NAA at the expansion stage of peach fruit and outlines the molecular mechanisms and signaling pathways (Fig. 9). In our study, we found that NAA treatment at the fruit expansion stage inhibited the growth and development of peach fruit by coordinating the content of IAA, ABA, and other hormones, but ethylene was not produced at this stage. Therefore, we speculated that ethylene was not the primary regulatory hormone of peach fruit at the early fruit growth stage, and the dual effect of NAA on the growth and development of peach fruit might be related to the changes in ethylene content. The application of auxin at the early stage of fruit development can delay peach fruit ripening, similar to the inhibitory effect of auxin on non-climacteric fruits; and the application of auxin at the later stage of fruit development can promote fruit ripening by inducing ethylene production. All in all, NAA treatment at the fruit expansion stage could inhibit some metabolism processes involved in the related genes in the growth and development of peach fruit by regulating multiple-hormone signaling networks. These results help reveal the short-term regulatory mechanism of auxin at the fruit expansion stage and provide new insights into the multi-hormone cascade regulatory network of fruit growth and development.

## Conclusion

At the fruit expansion stage, NAA treatment significantly increased the content of IAA and reduced the content of ABA by coordinating the plant hormone biosynthesis and signal transduction genes. Furthermore, NAA treatment down-regulated most of genes involved in the growth and development of peach fruit, including the cell wall metabolism-related genes (*PpEG*), sucrose metabolism-related genes (*PpSPS*), phenylalanine metabolism-related genes (*PpPAL, Pp4CL, and PpHCT*), and transcription factors (*PpNAC, PpMADS-box, PpDof, PpSBP, and PpHB*). Overall, we found that NAA treatment at the fruit expansion stage could inhibit some metabolism processes involved in the related genes in the growth and development of peach fruit by mediating multiple-hormone signaling networks.

## Materials and methods

### Plant materials and hormone treatments

Five-year-old ‘Xiaobaifeng’ peach trees were selected as the experimental material. The plant materials were grown under common field conditions at the National Peach Germplasm Repository in Nanjing, Jiangsu province, China (our partners, we have a long-term relationship). According to the growth and development curve of peach, fruit samples were collected at four stages: young fruit (20 days post anthesis, 20DPA), pit hardening (50 days post anthesis, 50DPA), expansion (70 days post anthesis, 70DPA), and mature stage (90 days post anthesis, 90DPA). Based on the results of preliminary experiments and horticultural practices [56], the fruits were soaked in 0.2 M 1-Naphthaleneacetic acid (NAA, with 1% Tween-80) and 0.2 M N-1-naphthylphthalamic acid (NPA, an auxin inhibitor, with 1% Tween-80) for 1 min at 70DPA, and control (CK) fruits were treated with clean water with 1% Tween-80 solution. The fruit samples were collected 24 h after treatment and contained three biological replicates. All of the fruit samples were separated into skin and flesh, and the flesh samples were immediately frozen in liquid nitrogen and stored at -80℃. Part of the flesh samples was used for hormone quantification analysis, and the other part was used for RNA-seq experiment.

### Hormone quantification analysis

Endogenous phytohormones, 3-indoleacetic acid (IAA), abscisic acid (ABA), methyl jasmonate (MeJA), gibberellin (GA), and trans-zeatin (ZT), were extracted from peach fruits as follows. Firstly, 200 mg of freeze-dried powder was extracted in methanol: water: formic acid (79: 20:1, v: v: v) for 12h at 4°C. After centrifugated, the supernatant was passed through the MAX SPE Cartridge (Waters, USA) and eluted with 1.25 M formic acid and 70% methanol. Next, the residue was redissolved using 0.5 mL of the extraction solution after the eluate had been rotary evaporated (Acetonitrile: formic acid: water= 5: 1: 94, v: v: v). Finally, the supernatant was filtered through a 0.22 μm organic filter and added to a chromatographic column. The content of plant hormones was measured using an ACQUITY UPLC H-Class System (Waters, USA). Chromatographic separation was conducted with an ACQUITY UPLC HSS T3 column (100 × 2.1mm, 1.7 um) and a binary solvent system of (A) Acetonitrilel (0.1% HCOOH) and (B) water (0.1% HCOOH). The gradient for buffer A was 5~70% for 1 min, 70~5% for 1 min, and 5% for 3 min, with a flow rate of 0.4 mL/min. All standards (≥ 98%) were purchased from Solarbio (Solarbio, Beijing, China). The content of plant hormones was analyzed in triplicate and calculated based on peak area measurements.

In addition, ethylene production was measured using a gas chromatograph [57]. Firstly, six peach fruits were placed in an airtight container for 2 h at 25 ± 0.5 °C. Then 1 mL of headspace gas was withdrawn from the chamber and injected into a gas chromatograph (Agilent 7890A, CA, USA), using helium as the carrier gas. Detector and injector temperatures were 220 ℃. Each measurement included three independent biological replicates.

### RNA extraction, cDNA library construction, and transcriptome sequencing

According to the manufacturer's instructions, total RNA was extracted from peach flesh using the RNAprep Pure Plant Kit (TianGen, Beijing, China). The concentration of RNA was measured by an Agilent 2100 Bioanalyzer (Agilent, USA), and the quality of RNA was checked by 1.5% agarose gel electrophoresis. Then, mRNA purification and cDNA library construction were performed with the Ultra II RNA Library Prep Kit for Illumina (NEB, USA). Finally, the qualified cDNA library was sequenced using an Illumina Novaseq6000 (Novogene Technology Co., Ltd, Beijing, China).

### Transcriptome data analysis

For high-quality clean reads, reads were filtered by removing reads containing adapter, Nbase, and low-quality reads. The clean reads were mapped to the peach reference genome (GCF_000346465.2) using HISAT2. For each transcription region, fragments Per Kilobase Million (FPKM) were calculated to quantify its transcript abundance using RSEM software. Differentially expressed genes (DEGs) were analyzed using the DESeq2 R package. P-value≤0.05 and |log2(Fold change)| ≥1 were set as the threshold for significantly differential expression. To obtain the biological function of DEGs, we performed enrichment analysis using Gene Ontology (GO) (http://geneontology.org/) and Kyoto Encyclopedia of Genes and Genomes (KEGG) (https://www.genome.jp/kegg/) databases [58, 59]. DEGs mapped in GO and KEGG pathways with a P-value of ≤0.05 were considered significantly enriched. All transcriptome data analysis and picture drawing were performed using the online platform NovoMagic (https://magic.novogene.com/).

### Validation of RNA-seq by qRT-PCR

To validate the accuracy of the RNA-Seq data, 16 DEGs were selected randomly for qRT-PCR verification. The PpTEF2 gene was used as an internal control for normalization, and the primers were shown in Supplementary Table S1. According to the manufacturer’s instruction, quantitative real-time (qRT)-PCR was performed using Hieff^®^ qPCR SYBR^®^ Green Master Mix (Yeasen, Shanghai, China) with the Applied Biosystems 7500 Real-Time PCR System (ABI, USA). The amplification system consisted of 10 μL of Hieff^®^ qPCR SYBR^®^ Green Master Mix, 0.8 μL of upstream and downstream primer, 2.0 μL of cDNA, and 6.4 μL of ddH2O in a total volume of 20 μL. Each PCR assay was carried out by three biological replicates. The relative expression level was calculated with the formula 2^-ΔΔCT^ = normalized expression ratio.

### Statistical analysis

The data processing and difference significance analysis were carried out using Excel 2019 and SPSS 22.0. Data were expressed as means ± SD. Comparisons between groups were performed by one-way ANOVA corrected with Tukey’s multiple comparison tests at a significance level of P < 0.05.

## Conclusions

At the fruit expansion stage, NAA treatment significantly increased the content of IAA and reduced the content of ABA by coordinating the plant hormone biosynthesis and signal transduction genes. Furthermore, NAA treatment down-regulated most of genes involved in the growth and development of peach fruit, including the cell wall metabolism-related genes (*PpEG*), sucrose metabolism-related genes (*PpSPS*), phenylalanine metabolism-related genes (*PpPAL*, *Pp4CL*, and *PpHCT*), and transcription factors (*PpNAC*, *PpMADS-box*, *PpDof*, *PpSBP*, and *PpHB*). Overall, we found that NAA treatment at the fruit expansion stage could inhibit the growth and development of peach fruit by mediating multiple-hormone signaling networks.

### Supplementary Information

Below is the link to the electronic supplementary material.

**Additional file 1: Table S1.** The Primers of DEGs for qRT-PCR analysis. **Table S2.** Summary of transcriptome data. **Table S3.** Statistics of the number of DEGs. **Table S4.** DEGs involved in auxin signal transduction and synthesis during peach fruit development. **Table S5.** DEGs involved in six plant hormone biosynthesis and signaling after NAA treatment. **Table S6.** DEGs involved in six plant hormone biosynthesis and signaling after NPA treatment. **Table S7.** DEGs involved in cell wall metabolism in response to auxin treatment. **Table S8.** DEGs involved in sucrose metabolism in response to auxin treatment. **Table S9.** DEGs involved in phenylalanine metabolism in response to auxin treatment. **Table S10.** TFs in response to auxin treatment.

**Additional file 2: Supplementary Figure S1.** Determination of the six hormones in response to NAA and NPA treatments. **Supplementary Figure S2.** Expression heatmap of sucrose metabolism genes in response to auxin treatment. **Supplementary Figure S3.** Determination of TFs in response to NAA and NPA treatments. Red represents the up-regulated TFs, blue represents the down-regulated TFs, and the vertical axis shows the number of TFs.

## Data Availability

All data are included in the manuscript, any additional information needed contact the corresponding author. The RNA-seq data have been deposited into the NCBI SRA under the accession number PRJNA992389 (https://www.ncbi.nlm.nih.gov/bioproject/PRJNA992389)
