# Supplementary material for: Exogenous auxin regulates the growth and development of peach fruit at the expansion stage by mediating multiple-hormone signaling
Source: BMC Plant Biol. 2023 Oct 18;23:499. doi: 10.1186/s12870-023-04514-2 (PMC10583367; doi:10.1186/s12870-023-04514-2)
Supplement: Supplementary file 2 — Additional file 2: Supplementary Figure S1. Determination of the six hormones in response to NAA and NPA treatments. Supplementary Figure S2. Expression heatmap of sucrose metabolism genes in response to auxin treatment. Supplementary Figure S3. Determination of TFs in response to NAA and NPA treatments. Red represents the up-regulated TFs, blue represents the down-regulated TFs, and the vertical axis shows the number of TFs. [file 12870_2023_4514_MOESM2_ESM.docx]

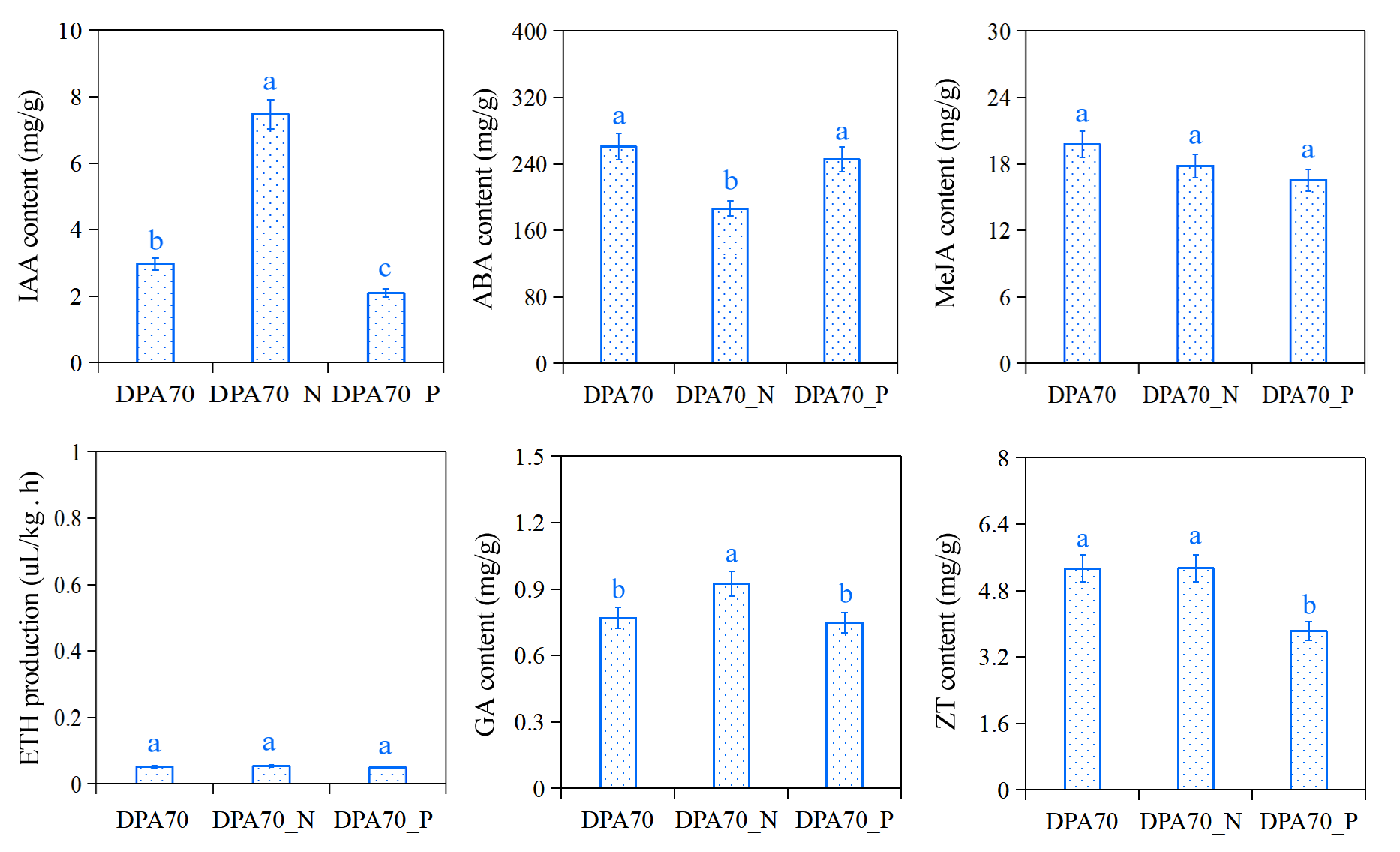


**Supplementary Figure S1.** Determination of the six hormones in response to NAA and NPA treatments


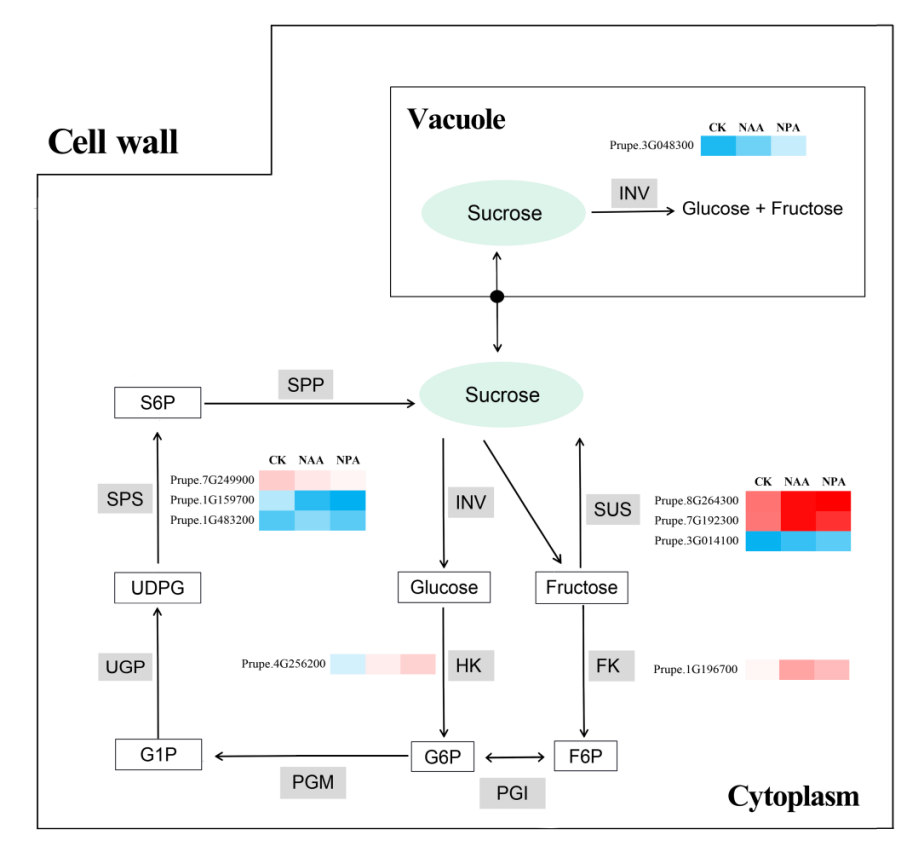


**Supplementary Figure S2.** Expression heatmap of sucrose metabolism genes in response to auxin treatment


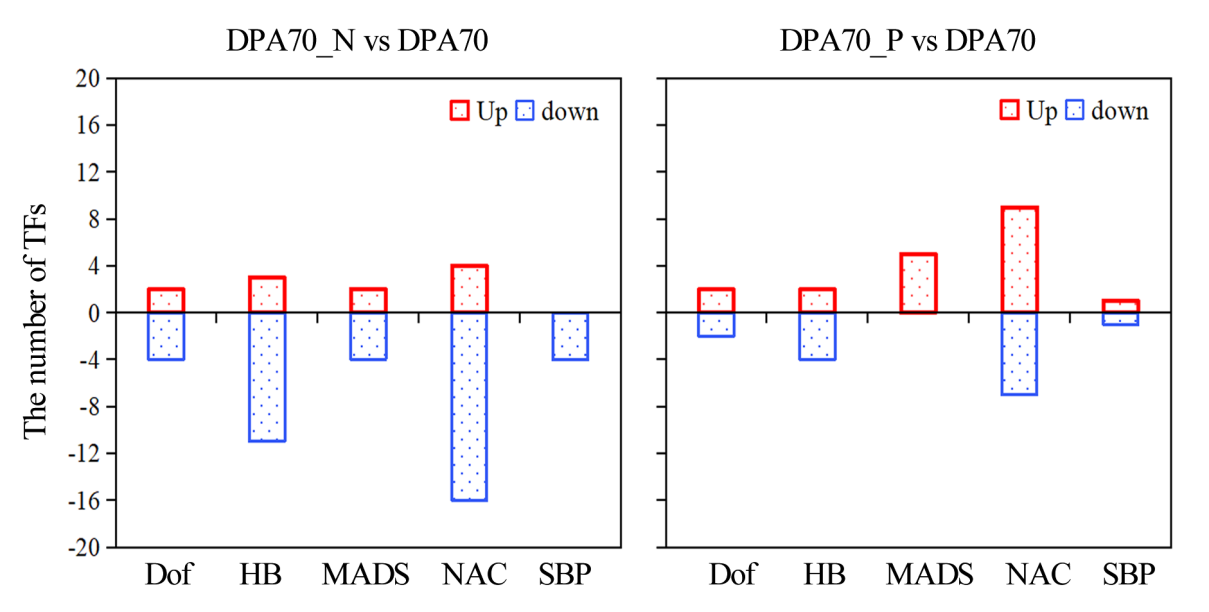


**Supplementary Figure S3** Determination of TFs in response to NAA and NPA treatments. Red represents the up-regulated TFs, blue represents the down-regulated TFs, and the vertical axis shows the number of TFs.
